# Reactions of an Isolable Dialkylsilylene with Aroyl Chlorides. A New Route to Aroylsilanes

**DOI:** 10.3390/molecules21101376

**Published:** 2016-10-15

**Authors:** Xu-Qiong Xiao, Xupeng Liu, Qiong Lu, Zhifang Li, Guoqiao Lai, Mitsuo Kira

**Affiliations:** Key Laboratory of Organosilicon Chemistry and Material Technology of Ministry of Education, Hangzhou Normal University, Hangzhou 311121, China; xqxiao@hznu.edu.cn (X.-Q.X.); lxpyoujigui@163.com (X.L.); ahbzmclq@163.com (Q.L.); gqlai@hznu.edu.cn (G.L.)

**Keywords:** dialkylsilylene, silyl ketone, acylsilane, insertion, X-ray analysis

## Abstract

The reactions of isolable dialkylsilylene **1** with aromatic acyl chlorides afforded aroylsilanes **3a**–**3c** exclusively. Aroylsilanes **3a**–**3c** were characterized by ^1^H-, ^13^C-, and ^29^Si-NMR spectroscopy, high-resolution mass spectrometry (HRMS), and single-crystal molecular structure analysis. The reaction mechanisms are discussed in comparison with related reaction of **1** with chloroalkanes and chlorosilanes.

## 1. Introduction

Acylsilanes or α-silyl ketones have been known as a unique class of silicon compounds [1,2,3,4,5,6,7,8,9], showing remarkably red–red shifted *n*→π* transition bands [1,2] and being useful as distinct reagents in organic synthesis [4,5,6,10,11,12,13,14,15,16,17,18,19]. Most of all, acyltris(trimethylsilyl)silanes are of particular importance, which were utilized for the synthesis of the first stable silicon–carbon doubly bonded compounds (silenes) [20,21]. However, the synthesis of acylsilanes is still limited because of the relatively facile silicon–carbon bond cleavage under the reaction conditions. The direct reaction of a silylmetal with an acyl halide afforded the corresponding acylsilanes, but the yields were usually low due to the undesired secondary reactions [22,23]. The oxidation of α-silyl alcohols using ordinary oxidizing reagents often leads to the corresponding aldehydes [5]. The first successful synthesis of an aroylsilane was achieved by using an elaborate two-step route in good yields (Equation (1)) [22], while it is not applicable for the synthesis of alkanoylsilanes.


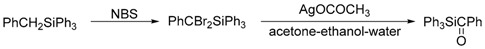
(1)

The dithiane route applicable for the synthesis of a wider range of acylsilanes was studied by Brook et al. [24] and Corey et al. [25] at the same time in 1967 (Equation (2)). The defect of the method is the use of a toxic mercury compound for the hydrolysis of the silylated dithianes.


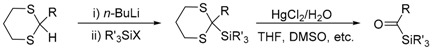
(2)

A variety of acylsilanes have been synthesized up to date using different methods, the reactions of protected aldehydes, esters, and other carboxylic acid derivatives, etc. with various silicon reagents [1,2,3,4,5,6,7,8,9].

During the course of our studies of the reactions of an isolable dialkylsilylene with various functional groups [26,27,28,29,30], we have found that the silylene inserts exclusively into the C–Cl bond of aroyl chlorides providing rather exceptional aroyl(chloro)silanes that cannot be obtained via conventional methods. Very recently, an acyl(halo)silane was utilized to synthesize an isolable silenyllithium (Equation (3)) [31].


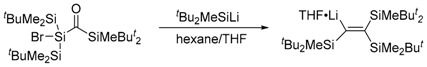
(3)

## 2. Results and Discussion

### 2.1. Synthesis and Characterization

The 1:1 reactions of dialkylsilylene **1** [32,33,34,35,36,37] with benzoyl and 4-substituted benzoyl chlorides **2a**–**2c** at −30 °C afforded the corresponding benzoyl(chloro)silanes **3a**–**3c** in high yields, indicating that the C(carbonyl)–Cl bond is much more reactive than the carbonyl group (Equation (4)) [38]. No significant difference was observed in the reactivity among benzoyl chlorides **2a**–**2c**. Even when an excess amount of **1** was used to a benzoyl chloride (2:1 mol ratio), the corresponding benzoyl(chloro)silane was obtained solely as the product. The expected reactions of **3** with silylene **1** would be prohibited due to the steric effects of bulky silylene moiety of **3**. The reactions of **1** with alkanoyl chlorides like acetyl chloride and butanoyl chloride afforded complex reaction mixtures. Because simple alkanoyl chlorides are more reactive than aroyl chlorides, the products of the reactions between **1** and the alkanoyl chlorides may react further with **1** to give the unidentified products.


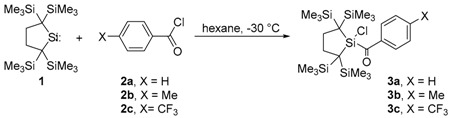
(4)

Benzoylsilanes **3a**–**3c**, which are stable thermally with definite melting points and under moist air, were characterized by ^1^H-, ^13^C-, and ^29^Si-NMR spectroscopy, high-resolution mass spectrometry (HRMS), and X-ray structure analyses.

### 2.2. NMR Spectroscopy

In the ^1^H-NMR spectra of **3a**–**3c**, two singlet signals due to four trimethylsilyl groups were observed in the region of 0.1–0.3 ppm [0.16, 0.30 (**3a**); 0.17, 0.29 (**3b**); 0.17, 0.29 (**3c**)], indicating that there are two types of trimethylsilyl groups because of their *C*s symmetry of **3**. In accord with the observation, two TMS carbon (δ ca. 3.2 and 4.3 ppm) and silicon signals (δ ca. 2.8 and 5.5 ppm) were observed in the ^13^C- and ^29^Si-NMR spectra of **3a**–**3c**. The signals at 225.3 (**3a**), 224.5 (**3b**), and 224.8 (**3c**) ppm in ^13^C-NMR spectra are ascribed to the carbonyl carbon signals, which are at higher field relative to the typical acylsilanes (ca. 240 ppm) [39,40]. However, these chemical shift values are significantly lower than those for typical ketones like benzophene (δ 196.7) and acetophenone (δ 198.2), indicating the unique electronic feature of acylsilanes. The ^29^Si-NMR resonances due to the ring silicon of **3a**–**3c** appear at the same chemical shifts of 27.8 ppm.

### 2.3. Molecular Structure Analysis

**Figure 1:**
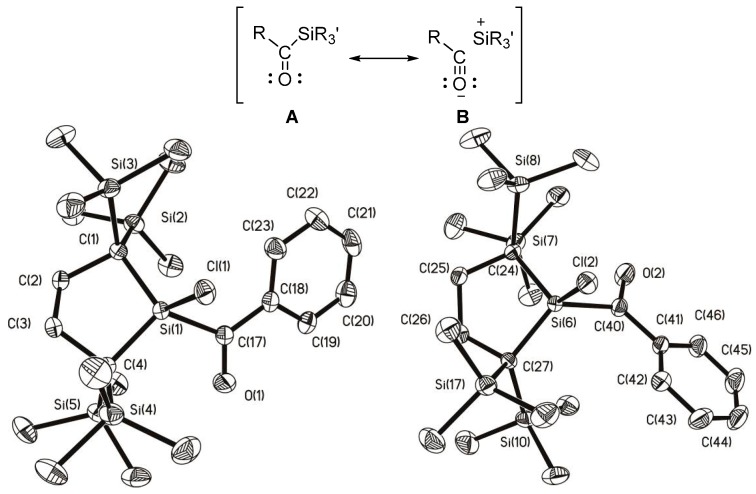
ORTEP drawing of the independent molecules in **3a**. (Hydrogen atoms are omitted for clarity. Thermal ellipsoids are shown at the 30% probability level.) Selected bond lengths (Å) and angles (°): Si(1)–C(4) 1.877(2), Si(1)–C(1) 1.890(2), Si(1)–C(17) 1.935(3), Si(1)–Cl(1) 2.1006(9), O(1)–C(17) 1.223(3), Si(6)–C(24) 1.882(2), Si(6)–C(27) 1.889(2), Si(6)–C(40) 1.929(2), Si(6)–Cl(2) 2.1001(9), O(2)–C(40) 1.227(3); C(4)–Si(1)–C(1) 102.55(11), C(17)–Si(1)–Cl(1) 95.66(9), O(1)–C(17)–C(18) 120.2(2), O(1)–C(17)–Si(1) 114.54(19), C(18)–C(17)–Si(1) 125.21(17), C(24)–Si(6)–C(27) 102.25(11), C(40)–Si(6)–Cl(2) 96.14(8), O(2)–C(40)–C(41) 119.4(2), O(2)–C(40)–Si(6) 114.64(18), C(41)–C(40)–Si(6) 125.89(17).

Molecular structures of compounds **3a**–**3c** were determined by X-ray single-crystal diffraction analysis. Yellow single crystals of **3a**–**3c** suitable for X-ray crystallography were obtained by slowly evaporating the solvent from their hexane solutions. The ORTEP drawing of compound **3a** is depicted in Figure 1. Compound **3a** was crystallized in space group *P*_-1_ with two crystallographically independent molecules in an asymmetric unit. The structural parameters of the two molecules in a unit cell are similar but different in the torsion angles of C(1)Si(1)C(17)O(1) and its equivalent, C(24)Si(6)C(40)O(2), (129.03° and 5.26°, respectively). The sum of bond angles around C(17) and C(40) are 360°, being in accord with the *sp*^2^ character of the carbonyl carbon atom. The distances of Si(1)–C(17) (1.935(3) Å) and Si(6)–C(40) bonds (1.929(2) Å), are significantly larger than the normal Si–C bond length (1.87–1.89 Å). A similarly long distance of the Si–C(carbonyl) bond (1.926 Å) has been observed in the molecular structure of acetyltriphenylsilane by Trotter et al. [41]. The origin may be ascribed to the effective σ(SiC)–*n*(O) conjugation as proposed by Ramsey, Brook, Bassindale, and Bock [42]. In other words, it is suggested that resonance form **B** contributed significantly to the bonding in acylsilanes.

**Figure 2:**
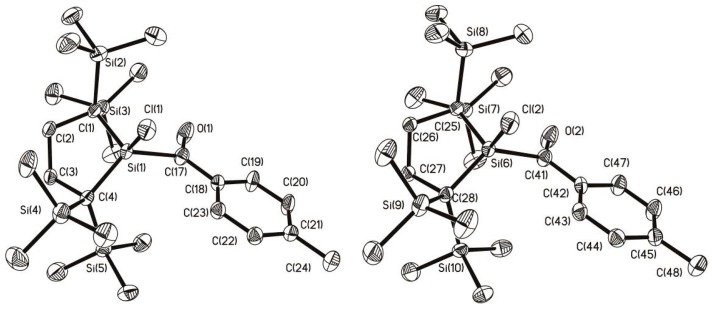
ORTEP drawing of the independent molecules in **3b**. (Hydrogen atoms are omitted for clarity. Thermal ellipsoids are shown at the 30% probability level.) Selected bond lengths (Å) and angles (°): Si(1)–C(1) 1.883(4), Si(1)–C(4) 1.898(4), Si(1)–C(17) 1.940(4), Si(1)–Cl(1) 2.1003(14), C(17)–O(1) 1.223(5), Si(6)–C(28) 1.888(4), Si(6)–C(25) 1.887(4), Si(6)–C(41) 1.927(4), Si(6)–Cl(2) 2.0967(15), C(41)–O(2) 1.216(5); C(1)–Si(1)–C(4) 102.26(17), C(17)–Si(1)–Cl(1) 97.97(13), O(1)–C(17)–C(18) 119.7(4), O(1)–C(17)–Si(1) 113.2(3), C(18)–C(17)–Si(1) 126.9(3), C(28)–Si(6)–C(25) 102.12(16), C(41)–Si(6)–Cl(2) 98.33(14), O(2)–C(41)–C(42) 119.1(4), O(2)–C(41)–Si(6) 114.4(3), C(42)–C(41)–Si(6) 126.3(3).

**Figure 3:**
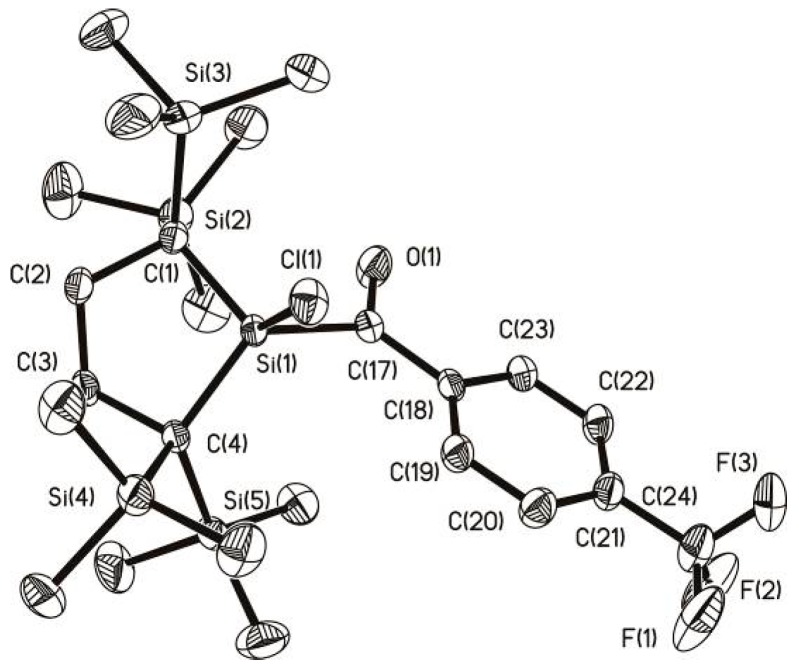
ORTEP drawing of compound **3c**. (Hydrogen atoms are omitted for clarity. Thermal ellipsoids are shown at the 30% probability level.) Selected bond lengths (Å) and angles (°): Si(1)–C(1) 1.8789(17), Si(1)–C(4) 1.8850(16), Si(1)–C(17) 1.9364(19), Si(1)–Cl(1) 2.1029(7), C(17)–O(1) 1.217(2); C(1)–Si(1)–C(4) 102.20(7), C(17)–Si(1)–Cl(1) 7.03(6), O(1)–C(17)–C(18) 18.54(16), O(1)–C(17)–Si(1) 15.19(14), C(18)–C(17)–Si(1) 126.03(13).

Similarly, compounds **3b** and **3c** were crystallized in space group *P21/n* and *P-1* and their molecular structures are shown in Figure 2 and Figure 3. A single crystal of **3b** has two crystallographically independent molecules in the asymmetric unit, while that of **3c** has one independent molecule. Their structural parameters are similar to those of **3a**.

### 2.4. Mechanistic Aspects

Because the insertion of silylene **1** into C–Cl [43,44,45,46,47,48] and Si–Cl [48,49,50,51,52,53] bonds have been reported, it would be desirable to propose the mechanisms of the present acylsilane formation as being consistent with the features of these precedents. The reactions of isolable dialkylsilylene **1** with chloroalkanes afford rather unusual product mixtures depending on the substrates. For example, **1** reacts with 1-chlorobutane to afford solely the corresponding butylchlorosilane 4, while the reaction of **1** with CCl_4_ gives only dichlorosilane 5 (Equation (5)) [43]. When cyclopropylmethyl chloride is used as a substrate, the rather unusual 2:1 adduct **6** was obtained in addition to **5** (Equation (6)) [43].


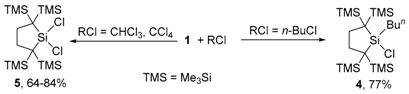
(5)


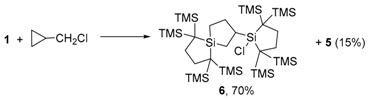
(6)

**Scheme 1:**
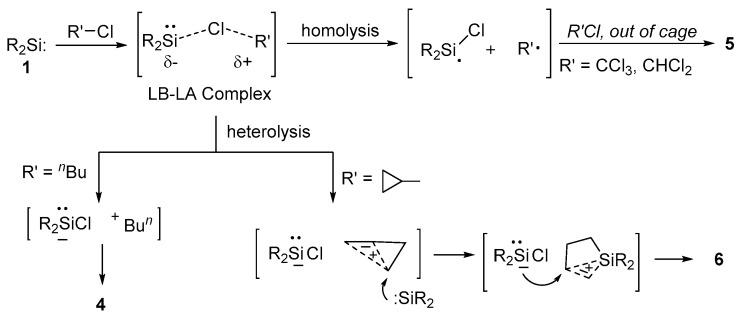
Mechanisms of the reactions of **1** with chloroalkanes.

The diverse modes of the reactions between **1** with chloroalkanes [43] suggest a complex nature of the mechanisms. The reactions may be understood uniformly starting from initially formed Lewis acid-base complexes as shown in Scheme 1. From the complex, ionic cleavage of the C–Cl bond followed by recombination would yield an alkylchlorosilane such as **4** [43]. The ionic mechanism is also applicable for the reaction of **1** with cyclopropylmethyl chloride, in which the intermediary cyclopropylmethyl cation or its equivalent 3-butenyl cation reacts with an extra silylene **1** forming 3-butenylsilyl cation and then finally **6**; the 3-butenylsilyl cation would be stabilized by the coordination of the terminal π bond. Chloroalkanes with less electron donating substituents like CHCl_3_ and CCl_4_ destabilize the carbocation intermediates and instead yield **5** after the homolysis of the C–Cl bond [54].

The aroylation of **1** may occur concertedly from the acylsilane-silylene complex as shown in Equation (7). Alternatively, the facile heterolysis of the C(carbonyl)–Cl bond from the complex followed by the coupling in cage may occur exclusively; the silylene serves as a Lewis acid to activate the C(carbonyl)–Cl bond (Equation (7)). The former concerted mechanism is preferred to the latter because of the similarity of the reactions with those of chlorislanes with **1** [50,51].


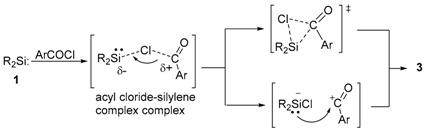
(7)

The insertion reactions of silylene **1** into the Si–Cl bonds of chlorosilanes have been found to occur cleanly [49,50]; hence, the concerted mechanism via three-membered cyclic transition states has been proposed. The mechanism has been supported by the detailed DFT calculations [55,56,57].


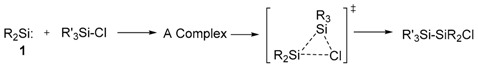
(8)

## 3. Materials and Methods

### 3.1. General Procedures

Manipulation of air-sensitive compounds was performed under a controlled dry argon atmosphere using standard Schlenk techniques. Tetrahydrofuran (THF), hexane, and toluene were distilled from sodium–benzophenone. All the other reagents were obtained from commercial suppliers and used without further purification. Dialkylsilylene **1** was prepared according to literature procedures [32]. ^1^H- (400 MHz), ^13^C- (100.6 MHz), and ^29^Si- (79.5 MHz) NMR spectra were recorded on a Bruker AV-400 spectrometer at room temperature (Bruker, Rheinstetten, Germany), using CDCl_3_ as the solvent. Melting points are uncorrected. High-resolution mass spectra (HRMS) were recorded on a Bruker Daltonics Apex-III spectrometer (Bruker, Rheinstetten, Germany).

### 3.2. Synthesis

#### 3.2.1. Synthesis of **3a**

A hexane solution of benzoyl chloride (0.45 g, 3.2 mmol) was added to a solution of dialkylsilylene **1** (1.12 g, 3.0 mmol) in hexane at −30 °C. The reaction mixture was allowed to stir for 2 h at 0 °C. The color of the solution changed from red to yellow. Then, the solvent was removed under vacuum. The resulting residue was purified by flash chromatography (Silica gel, 200–300 mesh; ethyl acetate/hexane, 1:300) to yield **3a** as a yellow solid. Yield: 0.98 g (64%). m.p. 152–154 °C; ^1^H-NMR (400 MHz, CDCl_3_): δ 8.07 (d, *J* = 7.2 Hz, 2H, *o*-Ar), 7.56 (t, *J* = 7.2 Hz, 1H, *p*-Ar), 7.49 (t, *J* = 7.2 Hz, 2H, *m*-Ar), 2.14 (s, 4H, C*H*_2_), 0.30 (s, 18H, Si*Me*_3_), 0.16 (s, 18H, Si*Me*_3_). ^13^C-NMR (101 MHz, CDCl_3_): δ 225.28 (*C*=O),138.90 (*C*_Ar_-C(O)),133.22 (*p*-Ar), 129.42 (*o*-Ar), 128.30 (*m*-Ar), 33.25 (*C*H_2_), 12.50 (*C*(SiMe_3_)_2_), 4.27 (Si*Me*_3_), 3.25 (Si*Me*_3_); ^29^Si-NMR (80 MHz, CDCl_3_): δ 27.84 (*Si*Cl), 5.53 (*Si*Me_3_), 2.85 (*Si*Me_3_); HRMS(ESI) calculated for C_23_H_45_ClOSi_5_: 513.2079, found 513.2078.

#### 3.2.2. Synthesis of **3b**

A hexane solution of *p*-methyl benzoyl chloride (0.49 g, 3.2 mmol) was added to a solution of dialkylsilylene **1** (1.12 g, 3.0 mmol) in hexane at −30 °C. The reaction mixture was allowed to stir for 2 h at 0 °C. The color of the solution changed from red to yellow. Then, the solvent was removed under vacuum. The resulting residue was purified by flash chromatography (Silica gel, 200–300 mesh; ethyl acetate/hexane, 1:300) to yield **3b** as a yellow solid. Yield: 0.97 g (61%). m.p. 174–177 °C; ^1^H-NMR (400 MHz, CDCl_3_): δ 7.96 (d, *J* = 7.2 Hz, 2H, *o*-Ar), 7.29 (d, *J* = 7.2 Hz, 2H, *m*-Ar), 2.43 (s, 3H, Ar-*Me*), 2.13 (s, 4H, C*H*_2_), 0.29 (s, 18H, Si*Me*_3_), 0.17 (s, 18H, Si*Me*_3_). ^13^C-NMR (101 MHz, CDCl_3_): δ 224.53 (*C*=O), 144.13 (*C*_Ar_-C(O)), 150.00, 136.63 (*p*-Ar), 129.59 (*o*-Ar), 128.98 (*m*-Ar), 33.24 (*C*H_2_), 21.77 (Ar-*Me*), 12.44 (*C*(SiMe_3_)_2_), 4.28 (Si*Me*_3_), 3.24 (Si*Me*_3_). ^29^Si-NMR (79 MHz, CDCl_3_): δ 27.84 (*Si*Cl), 5.47 (*Si*Me_3_), 2.81 (*Si*Me_3_); HRMS(ESI) calculated for C_24_H_47_ClOSi_5_: 527.2225, found 527.2235.

#### 3.2.3. Synthesis of **3c**

A hexane solution of *p*-trifluoromethyl benzoyl chloride (0.67 g, 3.2 mmol) was added to a solution of dialkylsilylene **1** (1.12 g, 3.0 mmol) in hexane at −30 °C. The reaction mixture was allowed to stir for 2 h at 0 °C. The color of the solution changed from red to yellow. Then, the solvent was removed under vacuum. The resulting residue was purified by flash chromatography (Silica gel, 200–300 mesh; ethyl acetate/hexane, 1:300) to yield **3c** as a yellow solid. Yield: 1.16 g, (67%). m.p. 160–163 °C; ^1^H-NMR (400 MHz, CDCl_3_): δ 8.17 (d, *J* = 7.2 Hz, 2H, *o*-Ar), 7.76 (d, *J* = 7.2 Hz, 2H, *m*-Ar), 2.16 (s, 4H, C*H*_2_), 0.29 (s, 18H, Si*Me*_3_), 0.17 (s, 18H, Si*Me*_3_). ^13^C-NMR (101 MHz, CDCl_3_): δ 224.78 (*C*=O), 140.87 (*C*_Ar_-C(O)), 134.31 (dd, *p*-Ar), 129.57 (*m*-Ar), 124.86 (*o*-Ar), 123.61 (dd, *J*_C-F_ = 271, *C*F_3_), 33.26 (*C*H_2_), 12.61 (*C*(SiMe_3_)_2_), 4.29 (*Si*Me_3_), 3.22 (*Si*Me_3_). ^29^Si-NMR (80 MHz, CDCl_3_) δ 27.83 (*Si*Cl), 5.66 (*Si*Me_3_), 2.94 (*Si*Me_3_); HRMS(ESI) calculated for C_24_H_44_ClF_3_OSi_5_: 581.1949, found 581.1952.

### 3.3. X-ray Crystallography

The diffraction data of **3a**–**3c** were collected on a Bruker Smart Apex II CCD diffractometer with graphite-monochromated Mo Kα radiation (λ = 0.71073 Å). All of the data were collected at ambient temperatures, and the structures were solved via the direct method and subsequently refined on *F*^2^ using full-matrix least-squares techniques (SHELXTL) [58]. Absorption corrections were applied empirically using the SADABS program [59]. The non-hydrogen atoms were refined anisotropically, and hydrogen atoms were located at calculated positions. A summary of the crystallographic data and selected experimental information is given in Table S1.

## 4. Conclusions

Isolable dialkylsilylene **1** was found to react with the C(carbonyl)–Cl bonds in aroyl chlorides **2** at low temperatures highly chemoselectively to give aroyl(chloro)silanes **3**; the carbonyl groups in neither **2** nor **3** react with silylene **1**. The structural analysis using NMR and X-ray crystallography indicate the lower field ^13^C-NMR resonance of the carbonyl carbon and longer Si–C(carbonyl) bond distance than the standard values. The facile and highly selective nature of the reactions suggests that the insertion occurs concertedly from the initial Lewis acid-base complexes, similarly to that of **1** into the Si–Cl bonds in chlorosilanes. We are hoping the present synthetic methodology is applicable in general for a wide variety of silylenes. The silylenes should be however relatively long-lived and their reactions with the aroyl chlorides should be fast enough to prevent their oligomerization. Further works on the acylsilanes with unique electronic properties are under progress in our laboratory.

## Supplementary Materials

Supplementary materials can be accessed at: http://www.mdpi.com/1420-3049/21/10/1376/s1. Crystallographic information for compounds **3a**–**3c** in CIF format and crystallographic tables.

## Appendix A

Crystallography data (excluding structure factors) for the structures reported in this paper have been deposited with the Cambridge Crystallographic Data Center, CCDC 979677 (**3a**), 979676 (**3b**) and 979675 (**3c**). Copies of these data can be obtained free of charge on application to the Director, CCDC, 12 Union Road, Cambridge CB2 1EZ, UK (fax: +44-1223-336033; e-mail: deposit@ccdc.cam.ac.uk or http:// www.http.ccdc.cam.ac.hk).
