# Supplementary material for: Reactions of an Isolable Dialkylsilylene with Aroyl Chlorides. A New Route to Aroylsilanes
Source: Molecules. 2016 Oct 15;21(10):1376. doi: 10.3390/molecules21101376 (PMC6273370; doi:10.3390/molecules21101376)
Supplement: Supplementary file 1 [file molecules-21-01376-s001.pdf]

Supplementary Materials: Reactions of an Isolable Dialkylsilylene with Aroyl Chlorides: A New Route to Aroylsilanes

Xu-Qiong Xiao, Xupeng Liu, Qiong Lu, Zhifang Li, Guoqiao Lai and Mitsuo Kira

Contents:

(1) NMR Spectra of **3a–3c**
(2) Table of crystallographic data for **3a–3c**

(1) NMR Spectra of 3a–3c

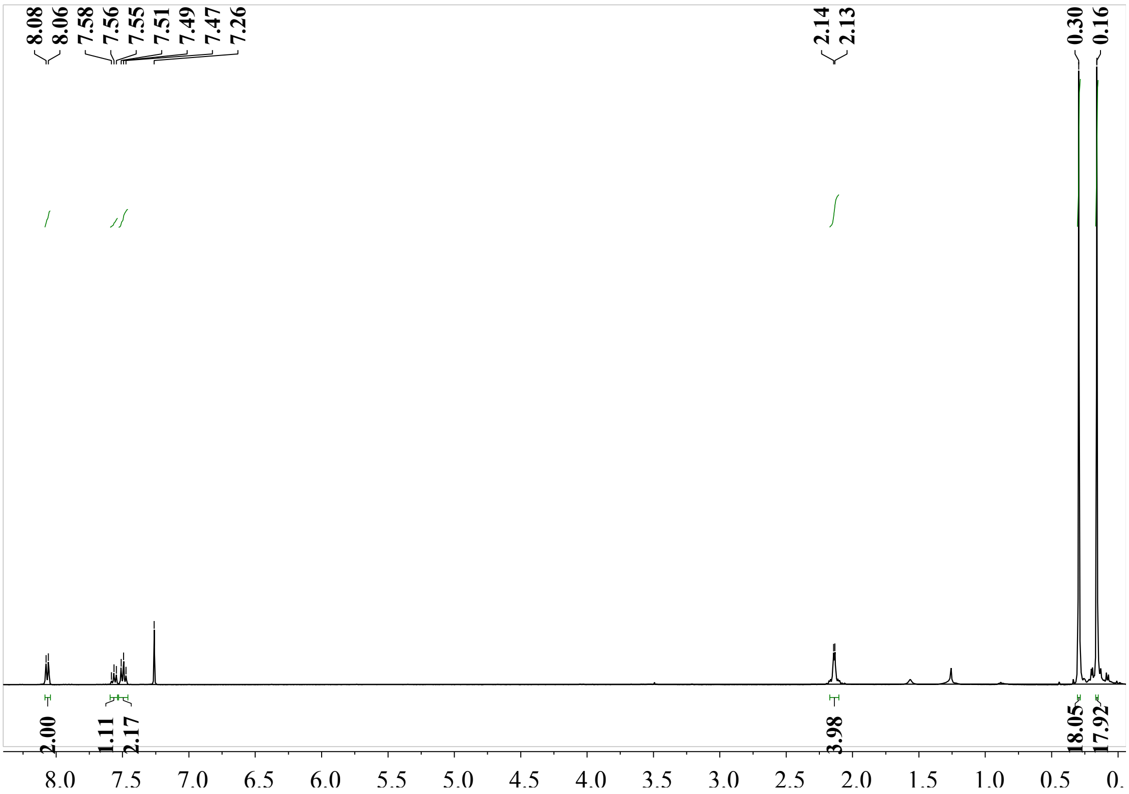

Figure S1. ^{1}H-NMR spectrum of **3a.**

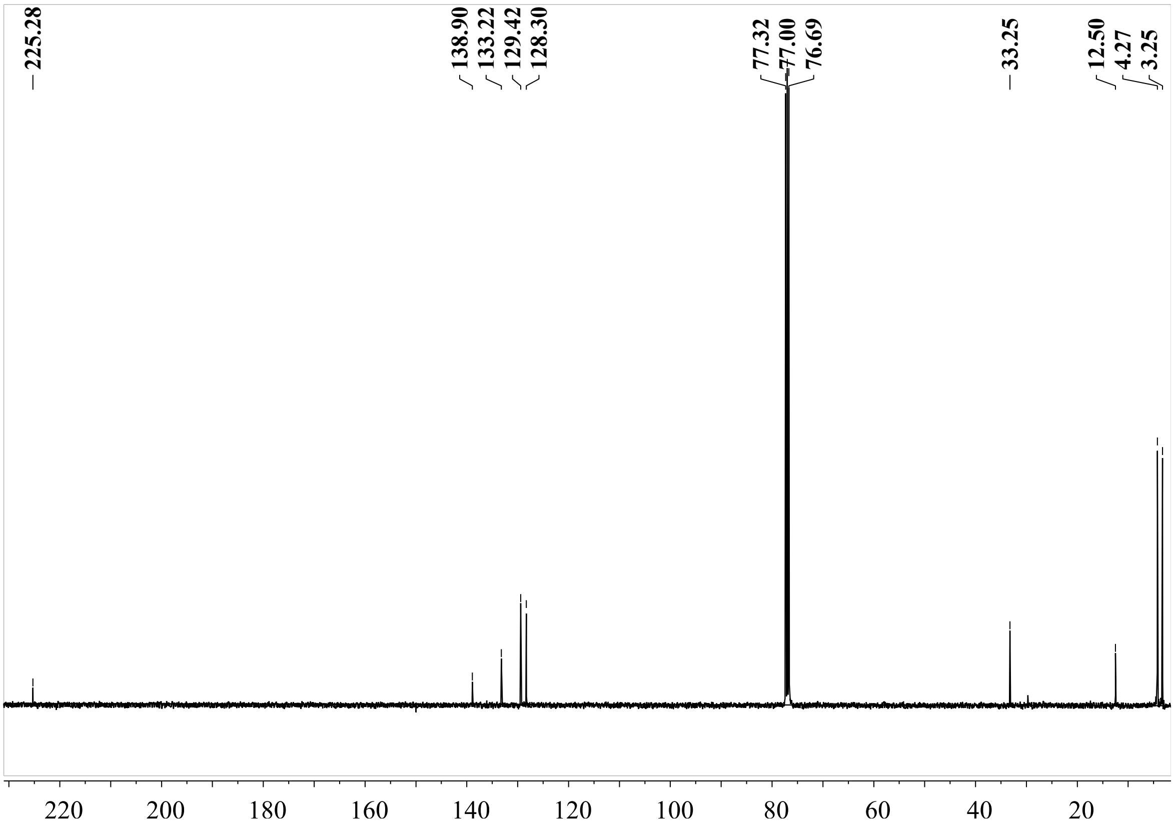

Figure S2. ^{13}C-NMR spectrum of **3a.**

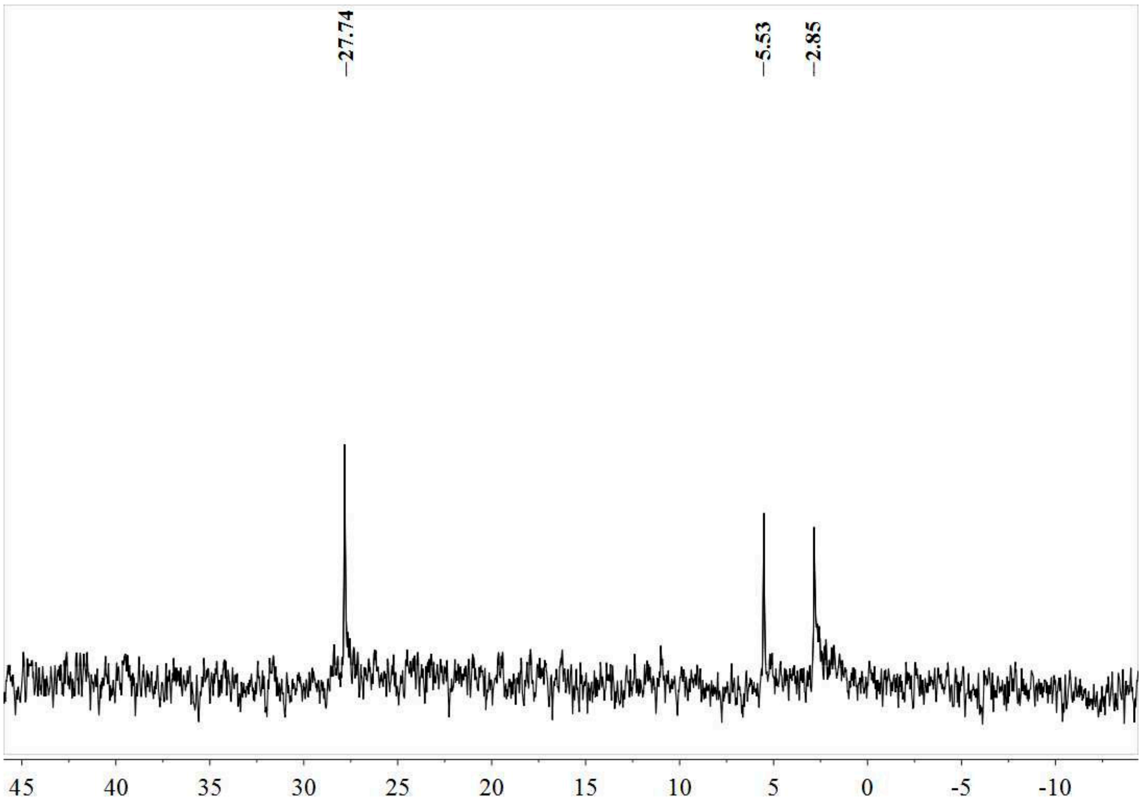

Figure S3. ^{29}Si-NMR spectrum of **3a.**

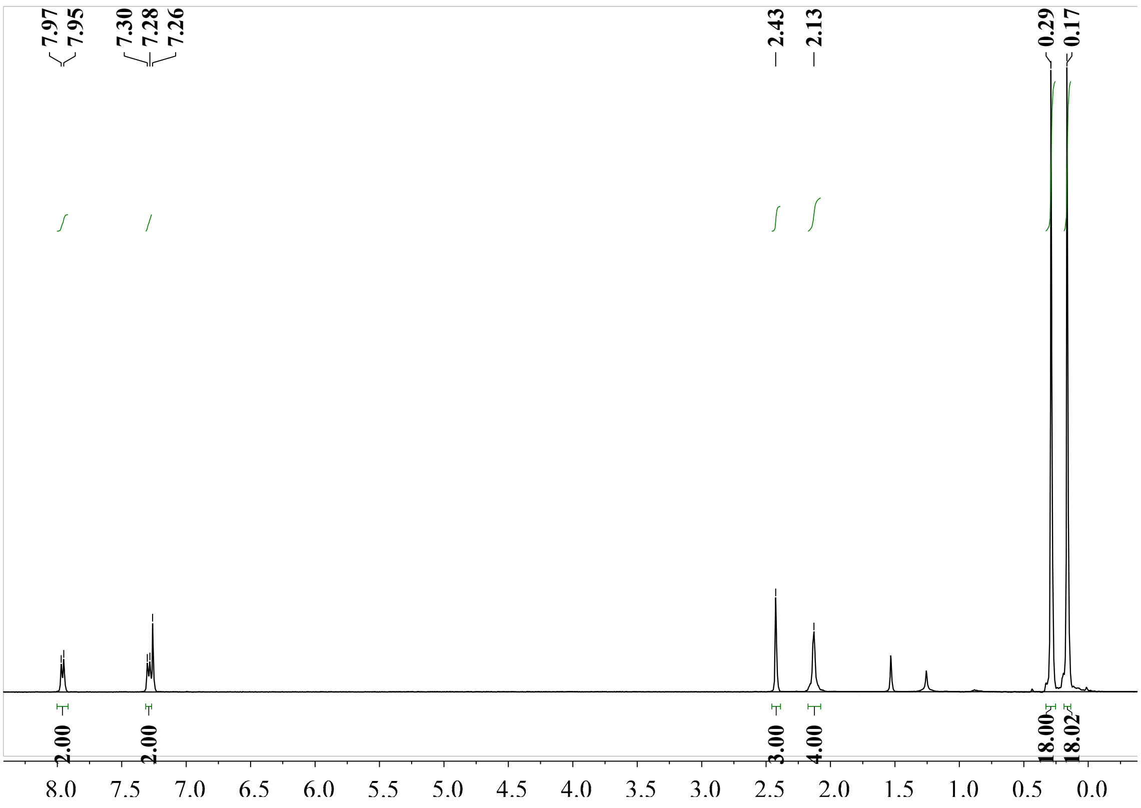

Figure S4. ^{1}H-NMR spectrum of **3b.**

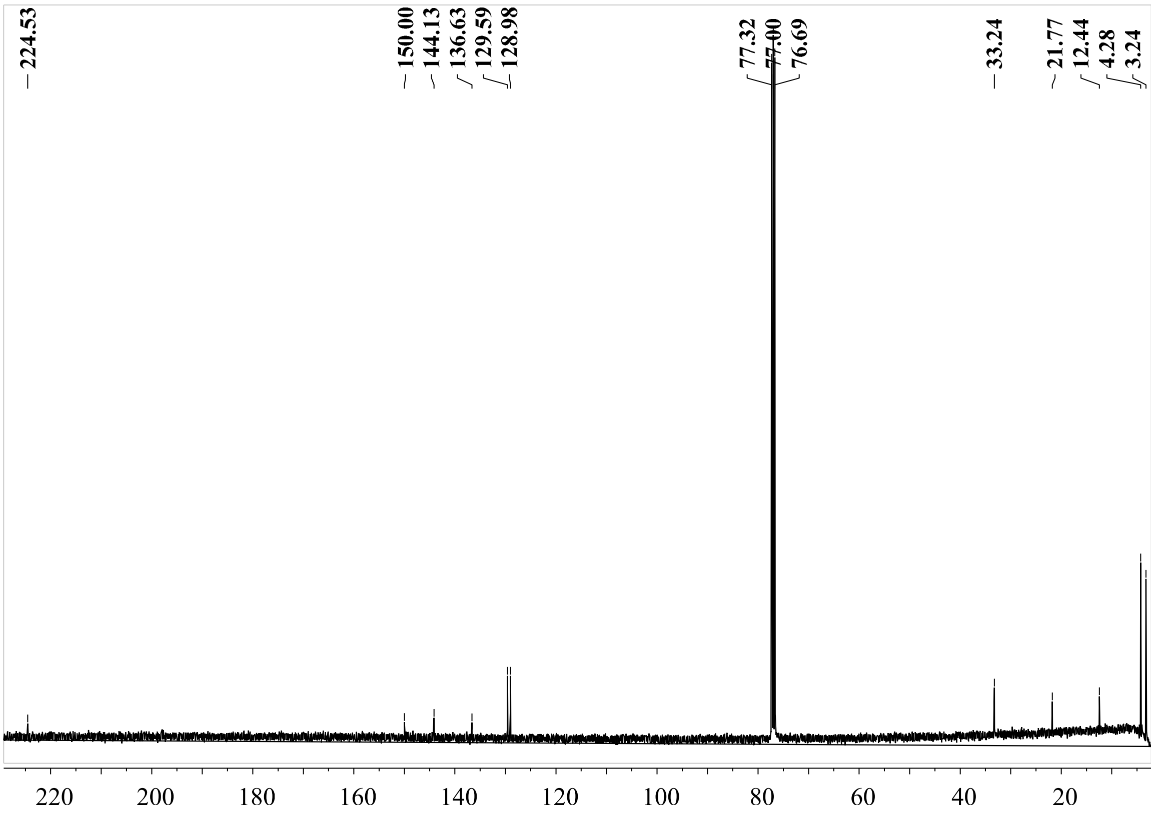

Figure S5. ^{13}C-NMR spectrum of **3b.**

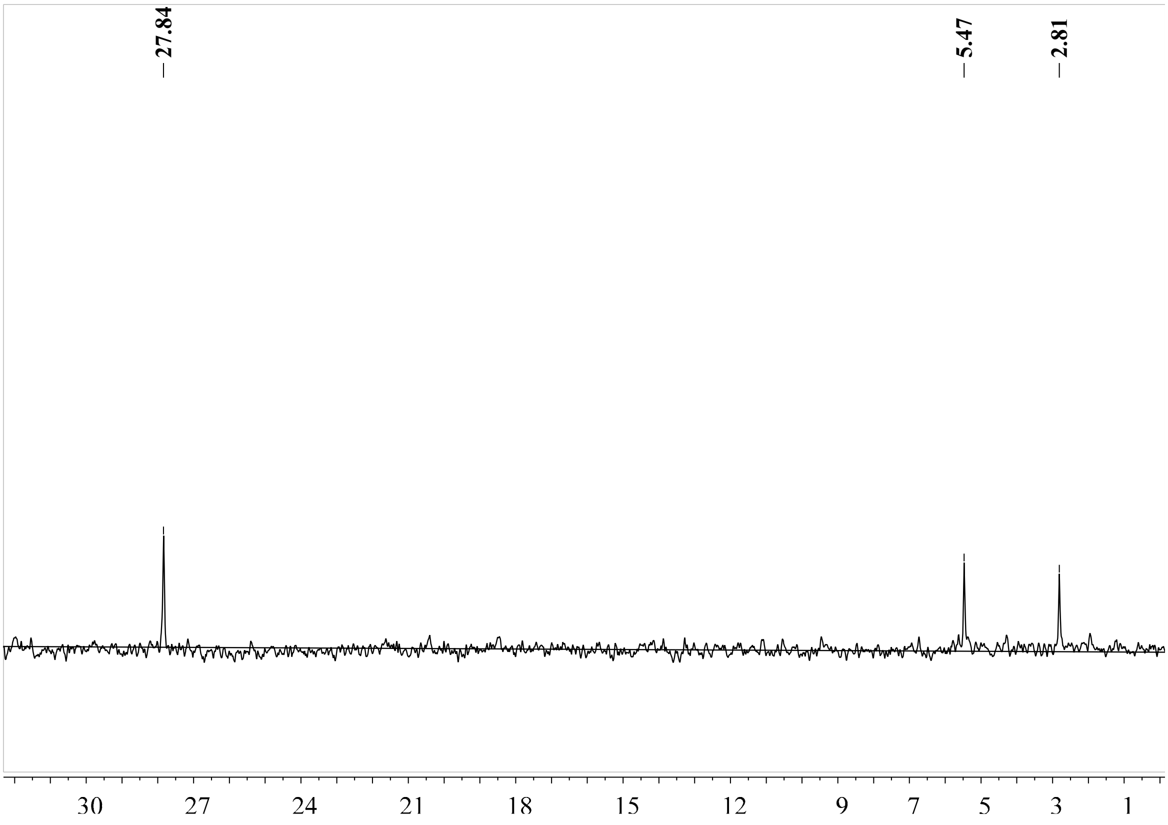

Figure S6. ^{29}Si-NMR spectrum of **3b.**

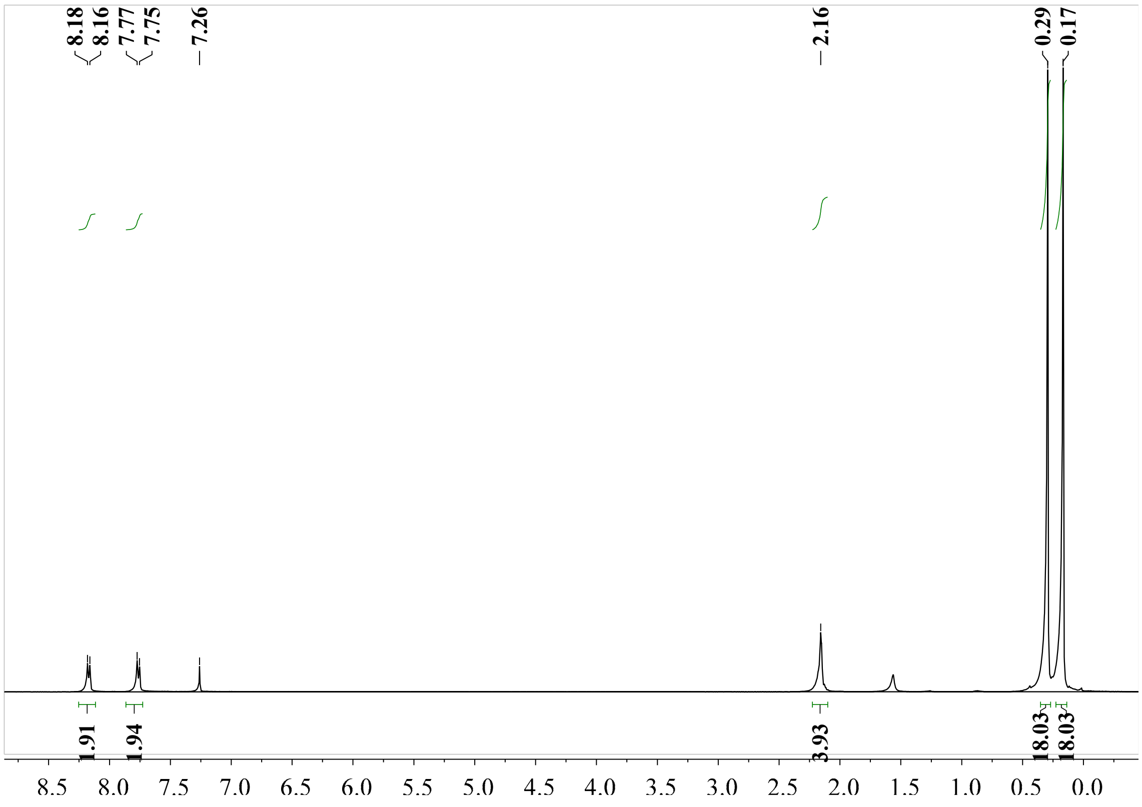

Figure S7. ^{1}H-NMR spectrum of **3c.**

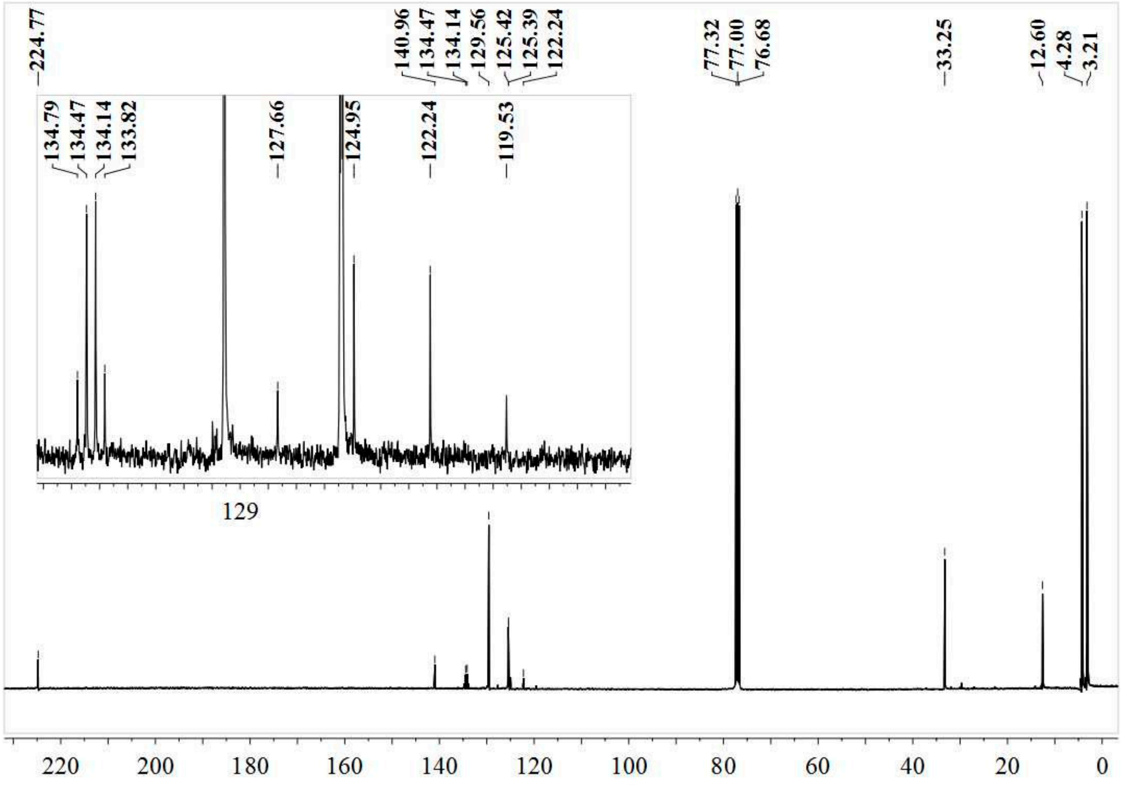

Figure S8. ^{13}C-NMR spectrum of **3c.**

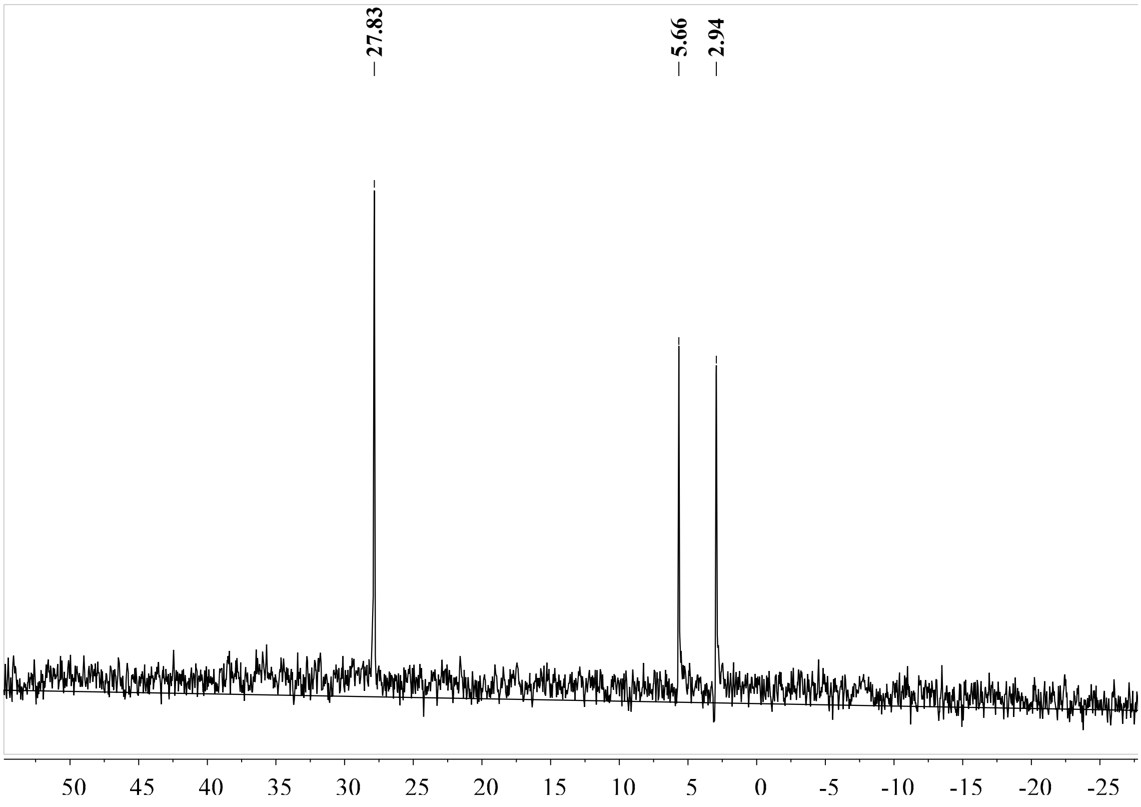

Figure S9. ^{29}Si-NMR spectrum of **3c.**

(2) Table of Crystallographic Data for 3a–3c

Table S1. Summary of Crystallographic Data for **3a**–**3c**.

Parameters	3a	3b	3c
Empirical formula	$C_{23}H_{45}ClOSi_5$	$C_{24}H_{47}ClOSi_5$	$C_{24}H_{44}ClF_3OSi_5$
Formula weight	513.49	527.52	581.49
Crystal system	Triclinic	Monoclinic	Triclinic
Space group	P-1	P2(1)/n	P-1
a (Å)	19.074(4)	17.2225(18)	9.1059(10)
b (Å)	15.060(4)	11.8089(12)	11.4480(13)
c (Å)	22.556(5)	30.911(3)	17.1330(19)
α (°)	90	90	72.731(2)
β (°)	112.076(5)	99.430(2)	88.767(2)
γ (°)	90	90	70.304(2)
V (Å^{-3})	6004(2)	6201.6(11)	1599.6(3)
Z, D_{calcd} (g·cm^{-3})	8, 1.136	8, 1.130	2, 1.207
μ (mm^{-1})	0.340	0.331	0.340
F (000)	2224	2288	620
Ref. collected	40,716	33,308	20,173
Independent reflections	13911	10850	7328
R(int)	0.0383	0.0448	0.0269
Completeness to θ (°)	27.73 (98.5%)	25.00 (99.4%)	27.57 (99.2%)
Max. and min. trans.	0.9048, 0.8483	0.7456, 0.6444	0.7456, 0.6596
GOOF	1.005	1.002	1.103
Final R indices	R_1 = 0.0455	R_1 = 0.0536	R_1 = 0.0400
$[I > 2\sigma(I)]$ [a]	wR_2 = 0.1200	wR_2 = 0.1621	wR_2 = 0.1125
R indices (all data)	R_1 = 0.04895	R_1 = 0.0949	R_1 = 0.0582
	wR_2 = 0.1528	wR_2 = 0.2361	wR_2 = 0.1280
$\Delta\rho_{max, min}$ (e. Å^{-3})	0.337, −0.275	0.563, −0.504	0.313, −0.231

[a] $R_1 = ||Fo| - |Fo||/|Fo|$; $wR_2 = [\Sigma w(F_o^2 - F_c^2)^2/\Sigma w|F_o^2|^2]^{1/2}$.
